# How Do Heterogeneous Networks Affect a Firm’s Innovation Performance? A Research Analysis Based on Clustering and Classification

**DOI:** 10.3390/e25111560

**Published:** 2023-11-19

**Authors:** Liping Zhang, Hanhui Qiu, Jinyi Chen, Wenhao Zhou, Hailin Li

**Affiliations:** 1 College of Business Administration, Huaqiao University, Quanzhou 362021, China; zhanglp@hqu.edu.cn (L.Z.); 2016411039@stu.hqu.edu.cn (H.Q.); 2116104003@stu.hqu.edu.cn (J.C.); wenhaoz2021@stu.hqu.edu.cn (W.Z.); 2 Research Center for Applied Statistics and Big Data, Huaqiao University, Xiamen 361021, China

**Keywords:** innovation performance, IUR collaboration network, IF collaboration network, decision rules, machine learning algorithms, entropy weight

## Abstract

Based on authorized patents of China’s artificial intelligence industry from 2013 to 2022, this paper constructs an Industry–University–Research institution (IUR) collaboration network and an Inter-Firm (IF) collaboration network and used the entropy weight method to take both the quantity and quality of patents into account to calculate the innovation performance of firms. Through the hierarchical clustering algorithm and classification and regression trees (CART) algorithm, in-depth analysis has been conducted on the intricate non-linear influence mechanisms between multiple variables and a firm’s innovation performance. The findings indicate the following: (1) Based on the network centrality (NC), structural hole (SH), collaboration breadth (CB), and collaboration depth (CD) of both IUR and IF collaboration networks, two types of focal firms are identified. (2) For different types of focal firms, the combinations of network characteristics affecting their innovation performance are various. (3) In the IUR collaboration network, focal firms with a wide range of heterogeneous collaborative partners can obtain high innovation performance. However, focal firms in the IF collaboration network can achieve the same aim by maintaining deep collaboration with other focal firms. This paper not only helps firms make scientific decisions for development but also provides valuable suggestions for government policymakers.

## 1. Introduction

Innovation has become the motor of firm growth and even national economic growth [1]. With the development of economic globalization, the core of competition among firms has changed from product price to innovation ability, which is the key source of the competitiveness of firms. However, in the context of the increasing difficulty and risk of innovation, merely focusing on traditional and closed innovation practices is insufficient for firms to cope with the accelerating technological convergence and rapidly changing market demands [2]. It is increasingly difficult for firms to carry out effective innovative practices only with their own resources and capabilities. Integrating external resources to carry out collaborative research and development has become an important strategy for firms [3,4]. Nowadays, collaboration networks formed by partnerships among firms are complex and play multiple roles in corporate governance and strategic decision making [5]. Patents record the whole process of technological innovation, which is practical to measure the innovation capacity of firms [6]. Building a collaboration network involving different innovation subjects based on collaborative patents is conducive to the flow and integration of innovation resources and is of positive significance for improving the efficiency of inter-organization collaborative innovation.

Collaboration networks are important ways for firms to obtain external innovative resources and knowledge. The impact of network characteristics on a firm’s innovation performance has been widely studied. Firstly, many scholars analyze the impact of network members’ position on their innovation performance from the perspective of network structural characteristics. For example, previous studies have proven that network centrality can have a positive or negative impact on a firm’s innovation performance [7,8,9], which inspires us to consider the differences in the impact mechanism between network positions and a firm’s innovation performance. Secondly, there have been some studies starting from the relationship characteristics of networks to analyze the impact of collaboration networks on a firm’s innovation performance. For example, Li et al. [10] believe that there is an inverted U-shaped relationship between the collaboration depth and innovation performance in industry–university–research collaboration. Zhang et al. [11] indicated that there is a similar relationship between collaboration breadth and a firm’s innovation performance. Thirdly, some scholars found that the impact of collaboration networks on a firm’s innovation performance varies with different partners and stages of collaboration [12].

A large number of studies have discussed the impact of an Industry–University–Research institution (IUR) collaboration network or Inter-Firm (IF) collaboration network on a firm’s innovation performance. Zhou et al. [13] found that the relationship characteristics of an IUR collaboration network have a more significant influence on a firm’s innovation performance than structural characteristics. Serge et al. [14] found that the impact of network structural characteristics on a firm’s innovation performance is complex in IF collaboration networks. Specifically, network centrality has a significant positive impact on a firm’s innovation performance while network density has a negative impact. The same variables can have different effects in different types of collaboration networks, but there are a few studies that have compared and analyzed the differences in the impacts of IUR and IF collaboration networks on a firm’s innovation performance. For example, the research of Zeng et al. [15] shows that IF collaboration networks can promote the innovation performance of SMEs better than IUR collaboration networks. However, because the research data are based on the views and experiences of respondents, the objectivity and universality of the research results are questionable. Therefore, it is necessary to compare the differences and similarities between the impacts of different types of collaboration on a firm’s innovation performance based on objective data.

Based on the existing research, we find that: (1) Previous studies have rarely noticed the differences in the impact of the heterogeneity of the collaboration networks of firms on their innovation performance. (2) Current research mainly focuses on a single variable in a collaboration network on a firm’s innovation performance, rarely paying attention to the combined impact of multiple variables on a firm’s innovation performance. (3) Many scholars analyze the linear relationship or simple non-linear relationship between network characteristics and a firm’s innovation performance rather than further thinking about the complex non-linear relationship between them. (4) The network characteristics of different types of focal firms may affect their collaboration mode and achievements. However, there have been few studies on how to improve a firm’s innovation performance based on different network characteristics.

To fill in the gaps of the previous research, this paper focuses on resolving the following issues:

(1) What are the similarities and differences between the characteristics of IF collaboration networks and IUR collaboration networks?
(2) Which characteristics play more significant roles in influencing IF collaboration networks and IUR collaboration networks?
(3) How do IF collaborative networks and IUR collaborative networks differ in terms of their paths influencing a firm’s innovation performance?

The main contributions of this paper are as follows: (1) The quality and quantity of patents are comprehensively considered through the entropy weight method to evaluate a firm’s innovation performance, which avoids using a single indicator and thus leading to incomplete research. (2) Machine learning algorithms including hierarchical clustering and classification and regression trees (CART) are used to analyze the complex non-linear influence mechanism between network characteristics and a firm’s innovation performance. (3) The influences of collaborative strategies chosen by focal firms of different collaboration networks on a firm’s innovation performance are analyzed. The conclusions of this paper will help firms understand their position advantages in a network and how to choose the right partners to improve innovation performance while avoiding innovation risks in the context of economic globalization.

## 2. Preliminaries

This section introduces the Social Network Analysis and machine learning algorithms mainly used in this study, which are Hierarchical Clustering and Decision Tree Classification.

### 2.1. Social Network Analysis

Social Network Analysis (SNA) is a method, used to study complex networks based on graph theory and mathematical models, that has been a key technology for analyzing collaborative networks [16]. SNA not only emphasizes that individuals are rational and that their actions are rational to make autonomous decisions but also notes that individuals are embedded in social networks and constrained by the structures formed by social networks. The core of the SNA method is the concept of “node-connection-relation”, where “node” refers to an individual or organization in the network, “connection” refers to the interaction between nodes, and “relation” refers to the characteristics of social relationships indicated by these connections. According to SNA, this paper analyzes the nodes, connections, and relations in networks and selects four variables from the perspective of network structure and relationship to describe the position characteristics of firms in collaboration networks. These are network centrality, structural hole, collaboration breadth, and collaboration depth.

### 2.2. Hierarchical Clustering

Clustering is a data mining algorithm that divides data objects with similar characteristics into clusters, based on a measurement of similarity, and divides dissimilar objects into different clusters [17], and the hierarchical clustering algorithm is a classical clustering method [18] that forms a tree-shaped clustering structure based on the distances between different data objects. Compared with other clustering algorithms, the hierarchical clustering algorithm has the following advantages. Firstly, the results of clustering do not differ depending on the preset number of clusters, so the results are objective. Secondly, the final tree-shaped clustering structure clearly shows the merging process of data objects, and it is easy to obtain the members of each cluster and the hierarchical relationships between clusters. This study uses the hierarchical clustering algorithm that adopts the bottom-up clustering strategy, which is known as the Agglomerative Nesting (AGNES) algorithm. Starting from forming a distinct cluster for each data object, clusters with the smallest distance are iteratively merged into larger and larger clusters until all data objects are in one cluster or a certain termination condition is met. For the principle and specific operation of the bottom-up hierarchical clustering algorithm, one can refer to Kaufman and Rousseeuw [19]. In this paper, focal firms with similar network characteristics are divided into the same cluster using this algorithm to further analyze how the configurations of different network characteristics impact a firm’s innovation performance.

### 2.3. Decision Tree Classification

The Decision Tree is a top-down classification algorithm based on tree structure. The root node contains all samples, the leaf nodes represent the final classification result, internal nodes represent the splitting criteria for splitting attributes and subsets, and the path from the root to each leaf node refers to the decision rule under this combination of conditional attributes [20]. Feature selection is the key to the decision tree algorithm; in this step, the current node is split by the optimal attribute selected by the appropriate indicator. Commonly used indicators are information gain and the Gini index; these two different indicators correspond to the two most famous decision tree algorithms: ID3 and CART [21]. Each of these three algorithms has advantages and disadvantages, and which algorithm to choose often depends on the specific data characteristics and application requirements. Information entropy describes the uncertainty of random variables and is often used in decision tree algorithms to measure data purity. Assuming that the proportion of class i samples in sample set D is $p_{i}(i=1,2,\ldots ,m)$, the information entropy of D is

(1) $$Ent\left(D\right)=-\sum_{i=1}^{m}p_{i}\log_{2}p_{i}$$

The higher the value of information entropy is, the lower the purity of data will be, which means that the proportion of all types of samples is close and it is difficult to distinguish. Information gain is used to measure the improvement in sample purity after a node is split by a selected attribute. If D is divided into several subsets {$D_{1},D_{2},\ldots ,D_{v}$}, and different weights are assigned according to the numbers of samples in these subsets, the entropy of D divided by attribute A is

(2) $${Ent}_{A}\left(D\right)=\sum_{j=1}^{v}\frac{\left|D_{v}\right|}{\left|D\right|}Ent(D_{v})$$

The information gain of attribute A is

(3) $$Gain\left(A\right)=Ent\left(D\right)-{Ent}_{A}\left(D\right)$$

The greater the information gain is, the greater the improvement in sample purity after node splitting will be, and the more the attribute should be taken as the splitting attribute of the current node for data classification. The ID3 algorithm selects features to divide samples according to information gain.

The CART algorithm uses the Gini index to measure the impurity of data as the basis of feature selection. The specific calculation formula is represented by the symbol of the above formula:

(4) $$Gini\left(D\right)=1-\sum_{i=1}^{m}{p_{i}}^{2}$$

Similarly, the Gini index of attribute A can be calculated by dividing D with attribute A as follows:

(5) $${Gini}_{A}\left(D\right)=\sum_{j=1}^{v}\frac{\left|D_{v}\right|}{\left|D\right|}Gini(D_{v})$$

The attribute that minimizes the Gini index after the sample set is partitioned will be the split attribute of the current node. It is noteworthy that no matter how many values a feature has, CART will sort and find an optimal segmentation point to divide the data into two parts without discretizing it. In a word, CART can handle continuous features well. The ID3 algorithm based on information entropy is only suitable for processing categorical data, while CART can process numerical data. Which algorithm to apply often depends on the types of variables and specific application requirements. Considering that all variables in this paper are continuous numerical values, and CART can divide the data into binary partitions to construct a classification tree, this paper selects the CART algorithm for classification. That is to say, taking the continuous network features as conditional attributes and the level of innovation performance of firms as the decision attribute, CART is employed to extract decision rules and analyze the influence of multiple variables on a firm’s innovation performance.

## 3. Framework and Data

This section constructs a research framework and introduces the process of patent data processing.

### 3.1. Research Framework

**Figure 1:**
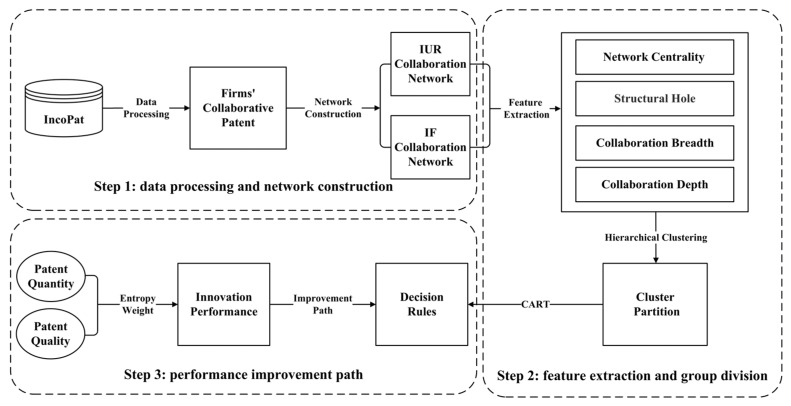
Research framework.

To analyze the impact of network characteristics of IF and IUR collaboration networks on a firm’s innovation performance, this study constructs a research framework based on machine learning algorithms. Considering the differences between firms, this study uses hierarchical clustering algorithm to analyze the impact mechanism of a firm’s innovation performance. And, the CART algorithm is employed to extract decision rules to analyze the combined impact of multiple factors on innovation performance. As shown in Figure 1, the research framework mainly consists of three steps. Step 1 is to process the patent data from the IncoPat database and further construct the IUR collaboration network and IF collaboration network. Step 2 is to extract features of the two collaboration networks including the collaboration breadth, collaboration depth, network centrality, and structural hole. Based on these features and the hierarchical clustering algorithm, focal firms are scientifically divided. Step 3 is to achieve the performance improvement paths of focal firms. Taking the collaboration breadth, collaboration depth, network centrality, and structural hole as conditional attributes and a firm’s innovation performance as the decision attribute, certain decision rules improving a firm’s innovation performance are obtained via the CART algorithm.

### 3.2. Data Sourcing and Processing

The artificial intelligence industry is prominent in the field of science and technology and engages in intensive innovation activities. It reflects the development trend of science and technology to a great extent. Authorized patents are not only publicly available but are also strictly checked and screened, which can reflect a firm’s capacity to innovate. This paper obtained a total of 252,657 authorized patents in China’s artificial intelligence industry during 2013–2022 from the IncoPat database. To ensure patent reliability, patents with the same or similar names are cleaned. Patents without firm-involved patentees are excluded. Drawing on the approach of Moaniba et al. [22], this paper defines patents with at least two patentees as collaborative patents. After that, 13,434 collaborative patents are left. To avoid sparse networks, firms with at least three collaborative patents are viewed as focal firms. Finally, 1145 focal firms in the IF collaboration network constructed by 7032 collaborative patents and 370 focal firms in the IUR collaboration network constructed by 2032 collaborative patents are obtained.

## 4. Variables and Measurement

In order to deeply analyze the impact of collaboration network characteristics on a firm’s innovation performance, the main variables and corresponding measures are introduced in this section.

### 4.1. Network Characteristic Variables

Chen et al. [23] found that the positions of innovation subjects in the network can reflect their access to resources, which has an important impact on innovation performance. Network centrality and structural hole are key indicators reflecting the position of firms in the collaboration network. Apart from the network position, network nodes encompass a wealth of additional information. This paper draws on the research of Kobarg et al. [24] and also selects collaboration breadth and collaboration depth as our variables. The following is a detailed introduction of these variables.

① *Network Centrality* (NC) describes the good interaction between a network member and other members, which can reflect the influence of a firm in the network. Generally, firms with high centrality in the network have better access to different information, resources, and the support of other network members [7], but those who are in less central network positions may not be able to acquire enough information, which is detrimental to a firm’s development [25]. Luo [26] believed that the closeness centrality can only be calculated when nodes of the network are connected in pairs. Therefore, in order to comprehensively and effectively evaluate the importance of nodes, referring to the practice of Qian [27], this paper integrates the degree centrality [4], betweenness centrality [28], and eigenvector centrality [29] into the variable network centrality (NC) using the Coefficient of Variation Method. The specific calculation formula of network centrality is

(6) $$v_{j}=\frac{n\sigma_{j}}{\sum_{i=1}^{n}x_{ij}},\;j=1,\;2,\;3$$

Here, $v_{j}$ and $\sigma_{j}$ refer to the coefficient of variation and standard deviation of each centrality index, respectively. $x_{ij}$ is the value of sample i on the jth centrality index. Then, the weight allocation mode of centrality is

(7) $$w_{j}=\frac{v_{j}}{\sum_{j=1}^{3}v_{j}}$$

Here, $w_{j}$ is the weight allocated to the jth centrality index. Therefore, the network centrality of sample i can be defined as

(8) $${Cen}_{i}=\sum_{j=1}^{3}w_{j}x_{ij}$$

② A *Structural Hole* (SH) acts as a “bridge” between nodes and can reflect the diversity of information received by firms in the network. Referring to the research of Guan et al. [30], the structural hole is defined as

(9) $$C_{i}=\sum_{j}{\left(p_{ij}+\sum_{q,q\neq i,q\neq j}p_{iq}p_{qj}\right)}^{2}$$

(10) $${SH}_{i}=2-C_{i}$$

Here, $p_{ij}$ is the direct relationship input between node i and node j, $p_{iq}$ is the intensity ratio of the relationship between node i and node q, and $p_{qj}$ refers to the intensity ratio of the relationship between node q and node j.

③ *Collaboration Breadth* (CB) refers to the number of partners a network member has, which can reflect the collaboration extent of firms in the collaboration network [31]. In particular, the measurement of collaboration breadth is different from that of degree centrality, which denotes the number of edges directly connected to a node in the network.

④ *Collaboration Depth* (CD) refers to the frequency of collaboration between a member and other members in the network, which can reflect the close degree of collaboration between firms and other innovation subjects in the network. Referring to the approach of Zhang et al. [28], this paper uses the ratio of the number of partners a firm has to the number of collaboration patents it owns as the measurement of collaboration depth.

### 4.2. Innovation Performance

Innovation performance (IP) is a comprehensive evaluation of a firm’s innovative activities and its achievements in products and services. Most research studies merely use the number of patents to measure innovation performance, while this paper comprehensively considers the quantity and quality of collaborative patents. Specifically, this paper selects five indicators including the number of claims, number of International Patent Classification (IPC) codes, citation times, number of inventors, and number of patentees of patents to measure patents’ quality combined with the entropy weight method (EWM), a method used to assign different weights to them according to the amount of information provided by each indicator. The number of claims is the basis for limiting the scope of patent protection [32]. The higher the number of claims is, the stronger the originality of the patent is and the higher the quality is. IPC is an internationally used tool for patent literature classification and retrieval [33]. The more IPC numbers a patent has, the more technical fields it covers and the more knowledge it contains. Citation times constitute an important index to measure the level of patent knowledge [34]. The number of patentees can indicate the wide range of applications and economic benefits of a patent. The greater the number of inventors is, the greater the difficulty of innovation will be, and the more technical resources and knowledge will be required [35]. In this paper, the EWM is used to combine the above five indicators into one indicator, which is an objective method to determine the weight of indicators according to the information entropy. Information entropy describes the uncertainty of random variables and reflects the diversity of data. The higher the information entropy value of a variable is, the more information it contains, and correspondingly, its weight in a series of variables is also higher. Referring to the practice of Li et al. [36], setting $x_{ij}$ (i = 1,2 … n, j = 1, 2, 3, 4, 5) as the value of the jth indicator of patent i, the information entropy of jth indicator is

(11) $$e_{j}=-\frac{1}{lnn}\sum_{i=1}^{n}\left(p_{ij}lnp_{ij}\right)$$

Here, $p_{ij}=\frac{x_{ij}}{\sum_{i=1}^{n}x_{ij}}$ and $x_{ij}\geq 0,\sum_{i=1}^{n}x_{ij}>0$. When $p_{ij}=0$, $\ln\left(p_{ij}\right)=0$. The specific calculation formula of the weight of the jth indicator is

(12) $$W_{j}=\frac{1-e_{j}}{\sum_{j=1}^{5}\left(1-e_{j}\right)}$$

The higher the value of $W_{j}\;is$, the higher the position of indicator j in patent quality is.

Thus, the innovation performance of patent i is

(13) $${\;Inno}_{i}=\sum_{j=1}^{5}x_{ij}W_{j}$$

The innovation performance of each patent is equally distributed to the patentees, so the innovation performance allocated to each patentee for this patent is

(14) $${\;AInno}_{i}=\frac{{Inno}_{i}}{m}$$

Here, m is the number of patentees of patent i.

Considering the number of patents, the innovation performance of firm k in the network is

(15) $$C_{k}=\sum_{1}^{q}{AInno}_{i}$$

Here, q is the total number of patents owned by firm k in the network.

## 5. Feature Analysis and Division of Firms

In this section, correlation analysis among variables is conducted to avoid possible severe collinearity and the characteristics of different collaboration networks are respectively analyzed. Meanwhile, focal firms with similar network characteristics are clustered using the hierarchical clustering algorithm.

### 5.1. Correlation Analysis

**Figure 2:**
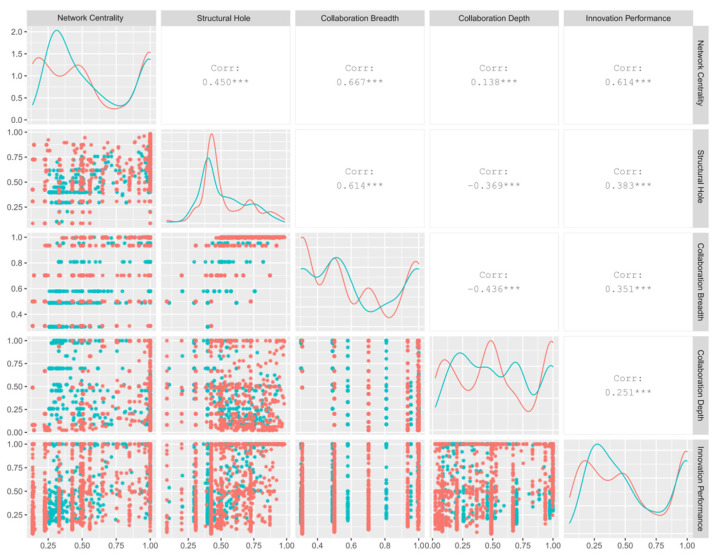
Correlation analysis of network characteristics and innovation performance.

In order to reduce the influence of uneven distribution and outliers of data on clustering, this paper uses the Cloud Model [37] to map data to membership degrees from 0 to 1 for calibration. With the purpose of preventing variable redundancy from affecting the reliability of research conclusions, the correlation among characteristic variables is further analyzed. Figure 2 presents the results of the correlation analysis. The red and blue dots and lines in the figure are respectively from the IF and IUR collaboration network. “*”, “* *”, and “* * *” indicate the significance level of 0.05, 0.01, and 0.001, respectively. The maximum correlation coefficient is 0.667, so there is no high correlation among variables [38]. In addition, the Variance Inflation Factor (VIF) of this paper is up to 3.863, and it can be considered that there is also no obvious multicollinearity among all variables. It can be inferred that in a complex network, a firm’s innovation performance is not simply determined by a single variable but is influenced by a combination of multiple variables.

### 5.2. Analysis of Network Characteristics

This paper analyzes the characteristics of network centrality (NC), structural hole (SH), collaboration breadth (CB), and collaboration depth (CD) and discusses their similarities and differences in IUR and IF collaboration networks. All these serve as the basis for constructing decision trees.

As illustrated in Table 1, both IUR and IF collaboration networks show obvious differences in innovation performance. The average innovation performance of the IUR collaboration network reaches 209.769 while the average innovation performance of the IF collaboration network is only 93.023, which is much lower than that of the IUR collaboration network. However, there is no significant difference between the proportions of the two networks achieving high levels of innovation performance.

**Figure 3:**
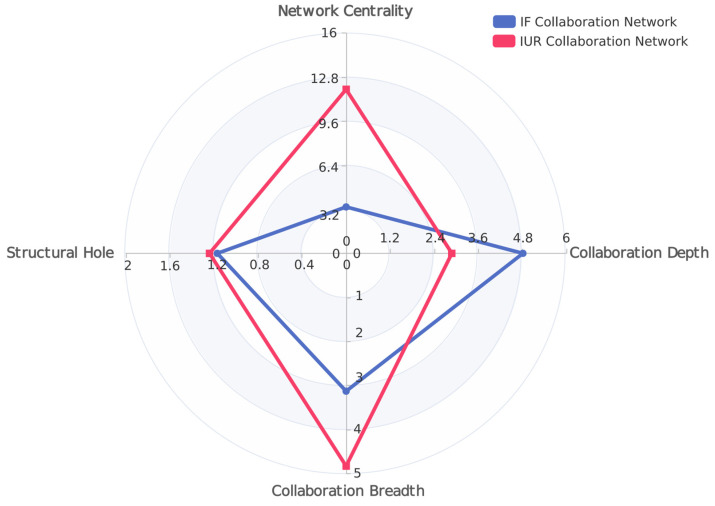
Radar map of NC, SH, CB, and CD, in IUR and IF collaboration networks.

As shown in Figure 3, the characteristics of both IUR and IF collaboration networks are quite different. First, collaboration breadth (CB) in the IF collaboration network is low while collaboration depth (CD) is high, indicating that in this network, focal firms tend to cooperate with other innovative subjects in the strategy of “less and better” and focus on the quality of partners. On the contrary, the higher collaboration breadth (CB) and lower collaboration depth (CD) in the IUR collaboration network indicate that focal firms are more willing to adopt the strategy of “wide and extensive” to obtain diverse knowledge. Next, there are obvious differences in the connection modes among the nodes of the two networks. The high network centrality (NC) in the IUR collaboration network reflects that there are more non-redundant and heterogeneous resources in this network. And, the values of structural hole (SH) in these two networks are similar, which means that the efficiency of information exchange and transmission are basically the same.

### 5.3. Division of Firms

This paper applies the hierarchical clustering algorithm to divide focal firms based on the computed network characteristics [38]. This helps in identifying the collaboration contexts of these firms and enables a better analysis of the innovation performance improvement paths for different types of firms.

Based on network centrality (NC), structural hole (SH), collaboration breadth (CB), and collaboration depth (CD), focal firms in each collaboration network are divided into two clusters. The average values of these characteristics are calculated for each cluster, which allows us to observe the differences between the two clusters.

As shown in Table 2, 204 focal firms in cluster I of the IUR collaboration network have a low possibility of obtaining high innovation performance. They are usually located at the edge of the network and are cautious about choosing partners. Such focal firms tend to focus their limited resources and energy on collaborating with certain important partners. A total of 166 focal firms in cluster II of the IUR collaboration network are more likely to obtain high innovation performance. They have high network centrality (NC) and structural hole (SH) values, which indicates that these focal firms have a greater voice in resource allocation.

Similar to cluster I of the IUR collaboration network, 654 focal firms in cluster I of the IF collaboration network have also lower innovation performance. They maintain deep collaboration with a very small number of firms. These focal firms have a high value in terms of collaboration depth (CD), but low values in terms of other characteristics. A total of 491 focal firms in cluster II of the IF collaboration network are more likely to obtain high innovation performance. Their network structural characteristics are at a medium level. They are usually close to the central position of the network and may have certain capabilities to integrate resources. Thus, they can maintain in-depth collaboration with more partners at the same time.

## 6. Analysis of Decision Rules

Decision tree algorithms vary based on different measurement indicators, such as information entropy and Gini index, when selecting variables to divide samples. In this paper, considering the continuity of variables and the efficiency of computation, the CART algorithm is ultimately employed to mine decision rules to analyze the improvement paths and configurations of different network characteristics with regard to a firm’s innovation performance. Support Degree (SupD) refers to the proportion of the sample size of the decision rule to the total number of samples in the cluster. Confidence Degree (ConD) represents the proportion of the number of samples supporting the final classification of the decision rule to the total sample size of this decision rule. This paper takes network centrality (NC), structural hole (SH), collaboration breadth (CB), and collaboration depth (CD) as conditional attributes and the discretized innovation performance as a decision attribute to respectively analyze the complex non-linear effects of multi-network characteristics on a firm’s innovation performance and extract decision rules for different clusters. The decision rules of a firm’s innovation performance are shown in Table 3. Next, we will conduct a specific analysis of the decision rules for a firm’s innovation performance in different clusters.

### 6.1. IUR Collaboration Network

**Figure 4:**
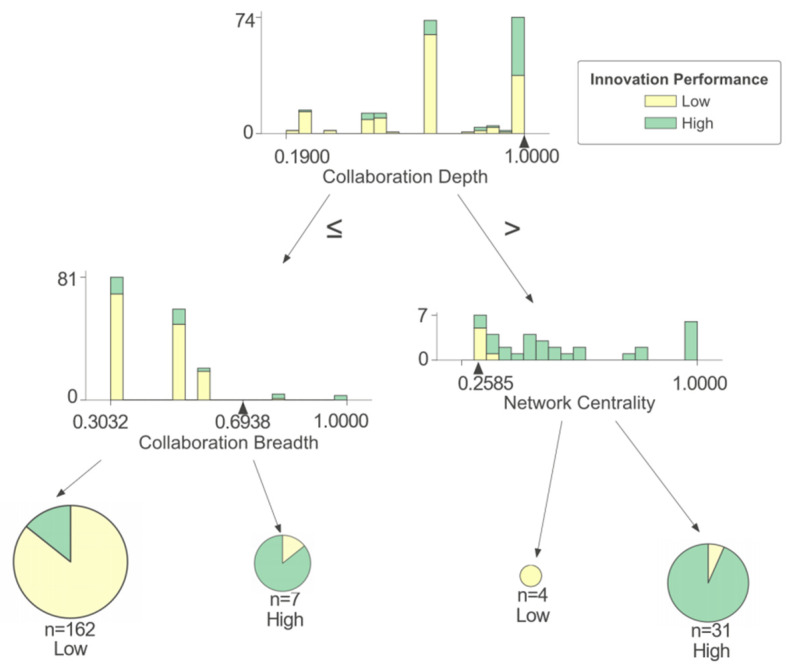
Cluster I of IUR collaboration network.

As shown in Figure 4, a firm’s innovation performance is mainly affected by collaboration depth (CD), collaboration breadth (CB), and network centrality (NC) in cluster I. There is a complex non-linear relationship between collaboration depth (CD), collaboration breadth (CB), network centrality (NC), and innovation performance. Collaboration depth (CD) positively affects a firm’s innovation performance. Specifically, when a firm has a high collaboration depth (CD), it is highly likely to achieve high innovation performance, while when the collaboration depth (CD) is low, most firms in cluster I can only obtain low innovation performance. Network centrality (NC) and collaboration breadth (CB) have a combined impact on a firm’s innovation performance, with collaboration depth (CD), to some extent. When collaboration depth (CD) is low, the higher the collaboration breadth (CB) is, the more it can help firms obtain high innovation performance, while firms with low collaboration depth (CD) and collaboration breadth (CB) are more likely to obtain low innovation performance. Existing research suggests that a higher collaboration breadth (CB) means that firms establish connections with a wider range of partners [24], which helps them gain more knowledge and resources and, to some extent, enhance their capability in terms of innovation practices. When collaboration depth (CD) is high, network centrality (NC) becomes more significant in influencing innovation performance. Firms with high network centrality (NC) usually have a strong ability to integrate resources and coordinate organizations in the network, which means they can easily obtain various resources from collaboration partners so as to grasp the advantages brought by deep collaboration and promote their collaboration projects [39]. Firms at the edge of the network often lack such resource-integrating and organization-coordinating abilities. On the contrary, they are forced to bear more communication costs and find it difficult to obtain sufficient innovation resources from partners, which is not conducive to their own output of innovation achievements [40].

For these firms, there is not only one combinational path to obtain high innovation performance. Firms can achieve high innovation performance by enhancing network centrality (NC) and collaboration breadth (CB). Even when the collaboration depth (CD) is low, the innovation performance can be improved by enhancing collaboration breadth (CB). In cases where their collaboration with universities and research institutions is not extensive, it would be advisable to allocate more resources and efforts to expand their collaborative network and increase the number of partners. By establishing connections with a wider variety of partners, including those from different types of organizations, firms can enhance their access to external resources and knowledge. However, if firms have already established highly trusted partnerships with universities and research institutions, they should focus more on maintaining existing relationships. By strengthening communication and developing new innovative projects, firms can enhance their influence and gradually become the core of the network so as to improve their innovation performance.

**Figure 5:**
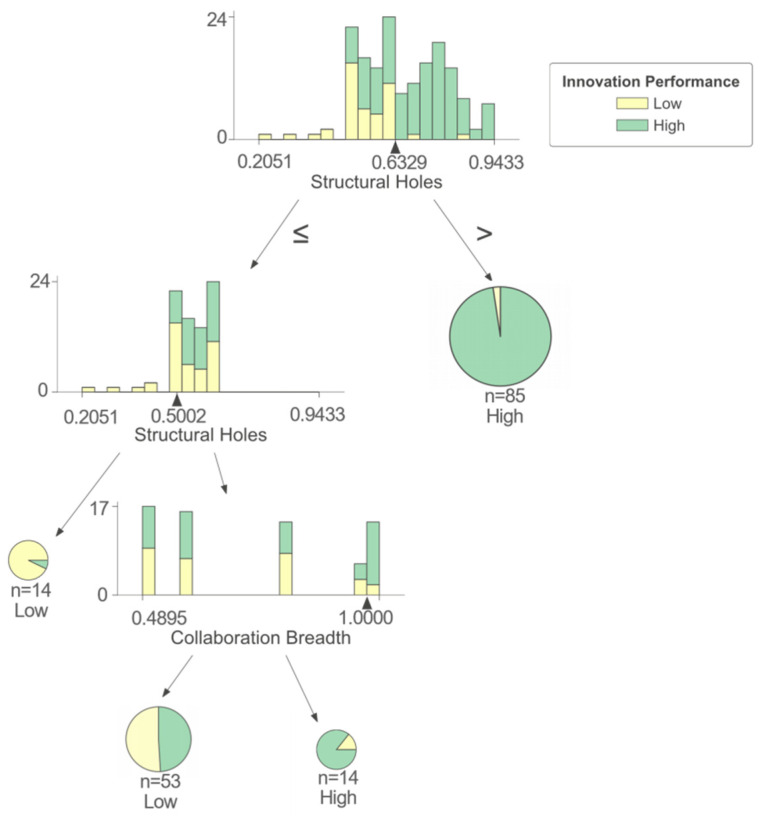
Cluster II of IUR collaboration network.

As shown in Figure 5, the structural hole (SH) and collaboration breadth (CB) have interactive effects on a firm’s innovation performance. Firstly, the structural hole (SH) has a direct influence on a firm’s innovation performance. When firms occupy a large number of structural holes in the network, they tend to obtain high innovation performance. When firms occupy fewer structural holes, their innovation performance is noticeably lower. This may be due to the role of the structural hole (SH) in enhancing information sharing and flow. When collaboration involves a wide range of organizations and institutions, there are inevitable barriers to information exchange and resource sharing. Occupying more structural holes can deepen collaboration between firms and partners, provide firms with more non-redundant resources, and thus improve the innovation capabilities of firms [11]. Secondly, when the number of structural holes occupied by firms reaches the average value, firms benefit from having a larger number of partners as they can acquire more information and resources to help formulate innovative strategies. On the contrary, firms will get limited information when having few partners, which will limit firms’ innovation practices.

For this cluster of firms, there are many combinational paths to obtain high innovation performance. Firms can improve the possibility of achieving high innovation performance by occupying more structural holes. When structural holes (SHs) are not enough, innovation performance can also be improved by strengthening the depth of collaboration. From the perspective of existing collaboration, it is most important for firms to enhance communication and facilitate the sharing of information, resources, and ideas. In addition, firms should actively pursue new partnership opportunities, particularly with organizations from diverse regions and fields, and refrain from information blocking, which hinders innovation performance.

### 6.2. IF Collaboration Network

**Figure 6:**
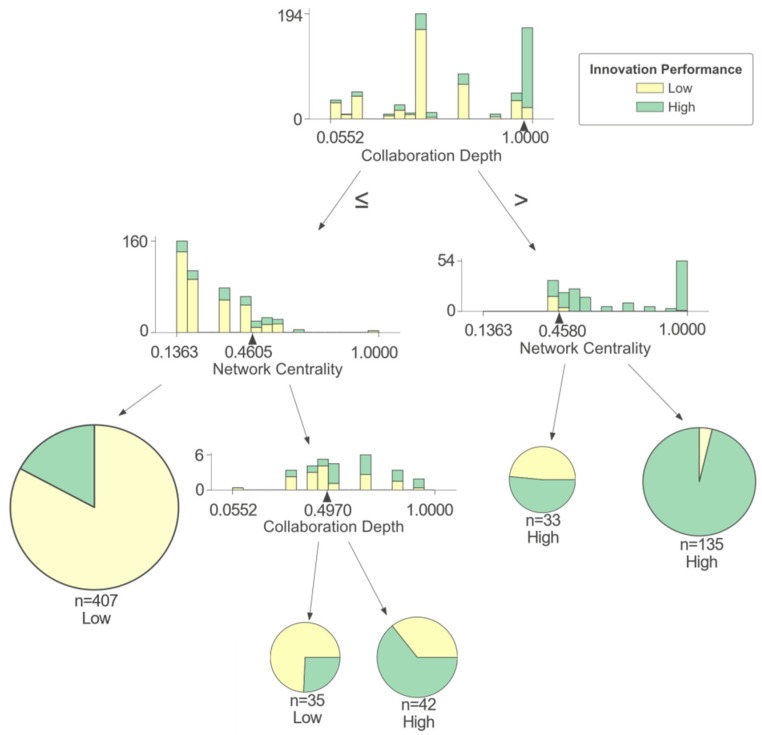
Cluster I of IF collaboration network.

As shown in Figure 6, in cluster I of the IF collaboration network, collaboration depth (CD) and network centrality (NC) have a complex interactive effect on a firm’s innovation performance. Obviously, collaboration depth (CD) has a greater impact on the innovation performance of firms. When the collaboration depth (CD) is higher, the firm is more likely to achieve high innovation performance. The lower the collaboration depth (CD) is, the lower the likelihood of a firm achieving high innovation performance is. Network centrality (NC) can compensate for the negative impact of low collaboration depth (CD) on a firm’s innovation. When both collaboration depth (CD) and network centrality (NC) are low, most firms tend to obtain low innovation performance. However, when firms have higher network centrality (NC), they can still generate high innovation performance, even without a significant advantage in collaboration depth (CD). Firms occupying the central position of the network often have stronger organizational ability. They act as coordinators in the collaboration process to ensure effective communication between partners and avoid the lack of trust caused by the lack of in-depth collaboration between firms, resulting in coordination and communication problems that delay the progression of collaboration projects [41]. It can be observed that more than half of the firms with high collaboration depth (CD) can still obtain high innovation performance with a low network centrality (NC). This finding differs from the conclusions drawn in cluster I of the IUR collaboration network. This could be due to the difference in collaboration strategies between the IUR collaboration network and IF collaboration network. Some scholars have found that IUR collaboration focuses more on the development of product performance [42] while IF collaboration emphasizes the novelty of products. This means that the innovation activities of IUR collaboration are concentrated in the early stages of research and development while the innovation activities of IF collaboration are concentrated in the later stages of commercialization. Therefore, compared to IUR collaboration, IF collaboration faces lower innovation risks. Even if firms cannot be in the core position of the network, they can still gather sufficient resources to apply the results of deep collaboration to innovation practices.

For this type of firms, the combinational paths to obtain high innovation performance mainly involve reasonably adjusting collaboration depth (CD) and network centrality (NC). While improving collaboration depth (CD), they should also improve their centrality in the collaboration network, which will increase the probability of achieving high innovation performance. Rather than maintaining contact with a large number of partners to cope with the uncertainty of innovative activity, these firms should prefer focused and specialized partnerships. They should maintain in-depth collaboration with other firms to share market information and, at the same time, strive to become leaders among their collaborative partners to ensure their core position in the network, which is an important measure to improve a firm’s innovation performance.

**Figure 7:**
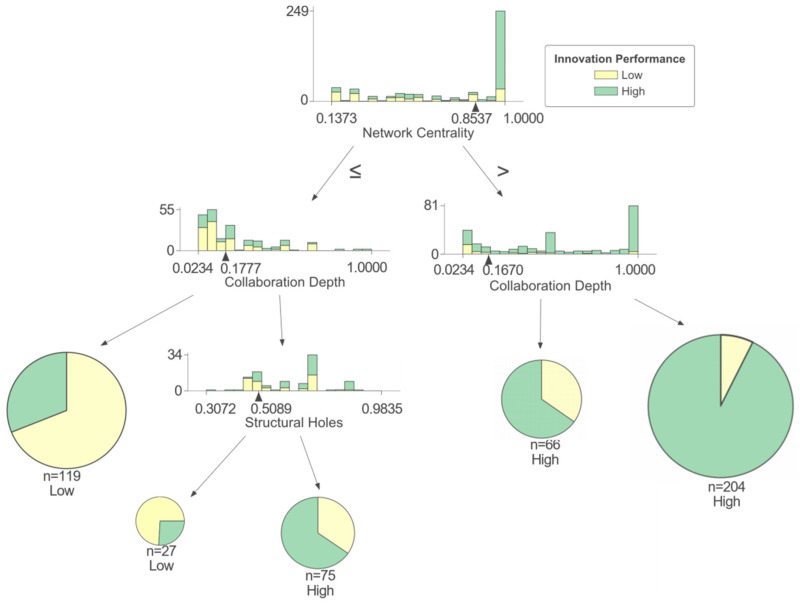
Cluster II of IF collaboration network.

As shown in Figure 7, a firm’s innovation performance is mainly affected by the network centrality (NC), collaboration depth (CD), and structural hole (SH) in cluster II. Specifically, network centrality (NC) directly affects the firm’s innovation performance. When network centrality (NC) is high, the possibility of achieving high innovation performance is very high. When network centrality (NC) is low, it is difficult for firms to obtain high innovation performance. However, the combination of the collaboration depth (CD) and structural hole (SH) can reduce the negative impact of low network centrality (NC) on a firm’s innovation performance. When network centrality (NC) is high, collaboration depth (CD) can effectively play the advantage of the firm’s core position in the network. Deep collaboration can promote the sharing of resources among firms, especially when firms are in the center of the collaboration network, it is conducive to the cultivation of trust among partners and thus improves the innovation performance of firms. When the network centrality (NC) of a firm is low, the number of structural holes can make up for the negative impact caused by a too-high collaboration depth (CD). When collaboration depth (CD) is high, the lack of structural holes is not conducive to the firm’s innovation performance, while having a large number of structural holes can help the firm maintain a certain innovation advantage. This finding is consistent with the research by Wu et al. [43]. If a firm occupies a larger number of structural holes, there are abundant opportunities for combining the knowledge elements possessed by the firm. Through deep collaboration, firms can explore the hidden rules between existing knowledge elements and build new connections to improve the level of innovation practice. On the contrary, when firms lack deep collaboration, structural holes provide opportunities for a wider selection of partners and disperse the risk of resource shortage caused by the lack of deep collaboration [44].

For firms in this cluster, the combinational paths to improve innovation performance mainly involve adjusting and optimize network centrality (NC), collaboration depth (CD), and structural hole (SH) values. Firms should improve their own network centrality (NC) as much as possible while not ignoring the promotion of collaboration depth (CD) and structural hole (SH) values to improve innovation performance. If they do not have a central position in the network, it is recommended to occupy as many structural holes as possible to expand access to information and innovative resources. If they occupy a central position in the network, they should concentrate resources and efforts on sustaining existing collaborative relationships and strengthening deep collaboration so as to transform the firm’s influence in the network into a competitive advantage and provide a strong guarantee for the firm’s innovation performance.

## 7. Conclusions and Discussion

### 7.1. Conclusions

This paper has analyzed the complex non-linear relationship between the network characteristics of focal firms and their innovation performance. Through machine learning algorithms [45] such as hierarchical clustering and the CART algorithm, the following conclusions have been obtained:

(1) The focal firm’s innovation performance is not determined by a single collaboration network characteristic but by a combination of several network characteristics. There is a complex non-linear relationship between network characteristics and a firm’s innovation performance, both in IUR and IF collaboration networks. By combining network centrality (NC), structural hole (SH), collaboration breadth (CB), and collaboration depth (CD), a firm can effectively modulate its influence on innovation performance.

(2) The focal firms in different collaboration networks adopt different collaboration strategies. In the IUR collaboration network, four network characteristics affect innovation performance in various combinations, and the two types of firms in the IUR collaboration network have different innovation performance improvement paths. Meanwhile, in the IF collaboration network, the improvement path of a firm’s innovation performance is mainly affected by network centrality (NC) and structural hole (SH).

(3) In addition to collaboration models, focal firms seeking to enhance their innovation performance should also consider the configuration of characteristics that align with their innovation capabilities. In the context of extensive collaboration, occupying a central position in the network often leads to significant breakthroughs in innovation activities. When focal firms occupy a central position in the network, it is important to make full use of the heterogeneous resources brought by structural holes and flexibly select collaborative partners to improve innovation performance. When the resources and energy of focal firms are not sufficient to sustain a large-scale collaboration network, improving the depth of collaboration with partners is an effective way to improve innovation performance.

### 7.2. Managerial Implications

This paper provides the following managerial implications for related firms and government departments:

(1) It is necessary for firms to seek and sustain close collaborative relationships with different partners when carrying out innovation practices. Firms should not be limited to just collaborating with other firms but should make full use of the think-tank resources of universities and research institutions to achieve innovation.

(2) Different types of firms should adopt different collaborative strategies. Firms should combine their own characteristics with their development goals to obtain continuous competitive advantages. For instance, in the realm of IUR collaboration, maintaining a broad network of partners is crucial, while in the context of IF collaboration, deep collaboration holds greater significance.

(3) Firms should improve their openness to adapt to the constantly changing external environment. By integrating the resources of partners and assimilating effective heterogeneous knowledge, firms can strengthen their ability to counter market risks.

(4) Government departments should establish a flexible incentive mechanism and guide different types of firms in choosing collaboration partners reasonably according to their own characteristics so as to improve their innovation capabilities and achieve economic development.

The main innovations of this paper are as follows: (1) Considering the complexity and comprehensiveness of innovation performance, this paper has taken into account both the quantity and quality of patents, using the entropy weight method, to explore the complex impact mechanism of innovation performance reasonably. (2) Machine learning algorithms such as hierarchical clustering and the CART algorithm have been introduced to analyze the innovation performance improvement paths for firms in different collaboration contexts so as to make conclusions more valuable for promotion. (3) The complex non-linear relationship between network characteristic variables and a firm’s innovation performance has been discussed, and certain suggestions that firms select collaborative partners have also been provided.

### 7.3. Limitations and Future Research

This paper provides some valuable implications for scholars, firms, and policymakers. However, there are still several limitations in this paper that should be overcome in future research. Firstly, the research sample used in this paper has been limited to authorized patent data obtained by China’s artificial intelligence industry. The numbers of patents and sample firms used in this paper are relatively small, which may impact the accuracy and validity of the research conclusions. Future studies can expand the sample size to include cross-industry and cross-country patent data to verify the conclusions of this paper on a broader scale. Secondly, although this paper has combined multiple variables to explore the complex non-linear relationship between network characteristics and a firm’s innovation performance, there are other important factors that can affect a firm’s innovation performance, include R&D investment, knowledge base, absorptive capacity, government subsidies, and environmental turbulence. Future studies can broaden the research perspective to include these internal and external factors and analyze their influence on a firm’s innovation performance. Thirdly, in future research, other machine learning methods can be used to further study relevant issues. This will help improve the depth of the research and the instructiveness and robustness of the conclusions and further enrich and expand the relevant research on the mechanisms affecting a firm’s innovation performance.

## Figures and Tables

**Table 1:** Descriptive statistics of innovation performance in IUR and IF collaboration networks.

Network	Average Innovation Performance	Proportion (%)	
IUR	209.769	High	48.6
		Low	51.4
IF	93.023	High	50.5
		Low	49.5

**Table 2:** The number of focal firms and the mean value of variables in each cluster.

Network	Cluster	Number	NC	SH	CB	CD	Innovation Performance	
							High (%)	Low (%)
IUR	I	204	3.528	1.004	1.961	4.067	28.4	71.6
	II	166	32.973	1.530	10.404	1.609	73.5	26.5
IF	I	654	1.556	0.973	1.532	5.745	38.7	61.3
	II	491	8.371	1.435	5.420	4.565	66.2	33.8

**Table 3:** Decision rules of firm’s innovation performance.

Network	Cluster	NC	SH	CB	CD	SupD (%)	ConD (%)	Decision Result
IUR	I	>0.311	-	-	>1.000	15.2	93.6	High
		≤0.311	-	-	>1.000	2.0	100.0	Low
		-	-	>0.694	≤1.000	3.4	85.7	High
		-	-	≤0.694	≤1.000	79.4	85.8	Low
	II	-	>0.633	-	-	51.2	97.7	High
		-	(0.500, 0.633]	>0.974	-	8.4	85.7	High
		-	(0.500, 0.633]	≤0.974	-	31.9	50.9	Low
		-	≤0.500	-	-	8.4	92.9	Low
IF	I	>0.458	-	-	>0.960	20.7	96.3	High
		≤0.458	-	-	>0.960	5.1	51.5	High
		>0.460	-	-	(0.497, 0.960]	6.4	64.3	High
		>0.460	-	-	≤0.497	5.4	74.3	Low
		≤0.460	-	-	≤0.960	62.5	82.9	Low
	II	>0.854	-	-	>0.167	41.6	92.7	High
		>0.854	-	-	≤0.167	13.4	65.2	High
		≤0.854	>0.509	-	>0.178	15.3	65.3	High
		≤0.854	≤0.509	-	>0.178	5.5	74.0	Low
		≤0.854	-	-	≤0.178	24.2	68.9	Low
